# Measure what matters: A survey-based examination of health equity tracking and measurement practices across healthcare systems in the United States

**DOI:** 10.1371/journal.pone.0323381

**Published:** 2025-05-21

**Authors:** Hallie Roth, Emilia De Marchis, Karyl Kopaskie, Alexandra Restall, Caroline Fichtenberg, Shaifali Ray, Kristen M.J. Azar

**Affiliations:** 1 Institute for Advancing Health Equity, Sutter Health, Walnut Creek, California, United States of America; 2 Department of Family and Community Medicine, University of California, San Francisco, California, United States of America; 3 Vizient, Incorporated, Chicago, Illinois, United States of America; 4 Social Interventions Research and Evaluation Network (SIREN), Center for Health and Community, University of California, San Francisco, California, United States of America; Wichita State University, UNITED STATES OF AMERICA

## Abstract

The objectives of this study were to understand how healthcare systems are incorporating equity into performance measurement and to uncover trends that inform healthcare systems’ efforts to advance equity. A national cross-sectional survey was designed and administered during Spring 2022 to evaluate organizational efforts to track and measure health equity. The survey examined clinical and non-clinical health equity metrics/indicators tracked at the executive-level. We identified variation in how health equity is measured. Of the 27 respondents, seven (25.9%) were in the planning phase, nine (33.3%) were in early implementation, seven (25.9%) had practices implemented for one to two years, and four (14.8%) had practices implemented for three or more years. Most systems were tracking clinical metrics and evaluating metrics across subpopulations. Metrics related to chronic disease management and preventive care were mentioned most frequently (23.6% and 16.0%, respectively). Race/ethnicity was the most utilized demographic filter to evaluate equity. Systems at later stages of implementation were tracking fewer metrics, yet many systems were still in early stages of implementation. Health systems need specific and pragmatic guidance to develop and implement equity measures tracked at the executive level. Insights from current health system initiatives can help inform guidelines from national quality organizations for disparity reduction in clinical outcomes.

## Introduction

Amidst widespread evidence, it is accepted that adverse social determinants of health lead to adverse health outcomes [1,2]. In response, healthcare systems have expanded their efforts to mitigate these risks and to identify and reduce healthcare disparities between populations. With an increased commitment to advancing health equity, many health systems have shifted strategic initiatives toward increasing diversity among clinicians, care teams and leadership, and renewing efforts to identify drivers of inequities and to mitigate them [3]. To date, there has also been increased attention to health equity from federal entities, like the Centers for Medicare & Medicaid Services (CMS) [4] and during the previous Biden-Harris administration [5]. As the healthcare industry evolves, the integration of health equity efforts within overall quality and patient safety programs and strategies will be crucial to long-term sustainability and institutionalization of these efforts.

Despite acknowledgement that disparities exist in health outcomes, and the momentum to advance health equity, health system efforts to eliminate disparities have by and large not been effective [3,6,7]. This is, in part, due to a lack of consensus on the best approach to identify and quantify equity gaps [8] which, in turn, hinders the ability to establish national benchmarks and performance standards. There are currently no evidence-based practices, nor consensus guidelines, for health systems to follow when identifying, tracking, and reporting health equity gaps among patient populations. Despite this, regulatory agencies, such as The Joint Commission, now require that organizations identify health care disparities in their patient populations using quality and safety data [9]. Other agencies, like CMS, have released recommended frameworks for systems to prioritize equity [4]. While this priority shift may catalyze action, the parameters are broad, allowing for a high degree of variation and the potential for ineffective practices [10]. Additionally, the impact of the new federal administration on health equity advancement is yet to be determined, which underscores the need to remain focused on defining health equity standards to guide health systems that remain dedicated to this work.

Current processes to measure and achieve equity performance goals, and corresponding reduction in health and healthcare disparities, can be examined to inform best practices and policies to implement across institutions. The development and deployment of consistent, standardized, and evidence-based measurement to address health equity across health systems will facilitate benchmarking and comparative performance analyses. These efforts can then facilitate the development of a broader accountability framework across health systems.

A cross-sectional survey was developed to examine variation in healthcare organizations’ practices related to measuring health equity, as well as barriers and facilitators to tracking health equity. This aim was achieved through an analysis of survey results.

## Materials and methods

A cross-sectional survey to assess healthcare systems’ efforts to advance health equity was designed by the Sutter Health Institute for Advancing Health Equity, in collaboration with Vizient’s Diversity, Health Equity, and Inclusion (DHEI) network, and researchers at the University of California, San Francisco. Vizient is a performance improvement company that provides cost, quality, and market performance solutions for more than 60% of acute hospitals and their affiliates across the nation. The survey intended to assess the current state of health system efforts around monitoring and measuring health equity at the executive-level and/or “C-Suite” level. Executive dashboards are business monitoring tools used by the Chief Executive Officer (CEO) and other health system leaders to monitor the organization’s most critical key performance indicators (KPIs). The survey was designed to assess overall healthcare system measurement of equity, as opposed to what individual member hospitals or affiliates may be independently measuring. Executive-level leadership and quality dashboards were chosen as the focus given the influence that these leaders have on health system decision-making and resource allocation. This work was formally assessed by the Sutter Health Institutional Review Board and found to be exempt from review as a quality improvement project. This work was undertaken to inform internal efforts to quantify, track and eliminate health disparities from the research institute’s hospital system. This paper is an analysis of de-identified data collected to facilitate those efforts. Thus, participant consent was waived.

The survey was administered from April 4, 2022 to June 6, 2022 to network members. An invitation to participate was sent via email to designated health equity leaders within all U.S. health systems registered with Vizient’s DHEI network (131 organizations represented). Membership is comprised of health system leaders with a focus on health equity practices within their respective organizations. The survey was completely voluntary, and no incentives were provided for participation. Participants were asked to coordinate internally to complete and submit one survey per health system. Data were collected via self-reported participant responses entered into a secure REDCap form managed by the research institute team [11]. In instances where the organizational structure contained more than one hospital or affiliate with different practices, participants were asked to report responses at the system level.

The survey included six sections (S1 Appendix). Section I solicited basic information on the responding organization. Region was assigned using Census Bureau-designated regions [12]. Number of hospitals in the system was assigned using the 2021 Agency for Healthcare Research and Quality (AHRQ) Compendium of U.S. Health Systems [13]. Section II queried the use of executive-level monitoring and reporting practices. Participants were asked to categorize their current practices for equity tracking into one of four tracking phases. Section III collected qualitative data on individual health equity indicators/metrics being tracked, and corresponding targets/benchmarks, through free-form text fields. This section also solicited information on sociodemographic “filters” (parameters) used across metrics by which to evaluate disparities. For example, if a health system is tracking the colorectal cancer screening rate of their patient population (metric) they may choose to view the data by race/ethnicity or age (parameters). Section IV asked for information to assess how health systems were measuring their performance against these metrics. Section V assessed additional monitoring practices, including the frequency of reporting. Section VI collected qualitative, free-form text responses related to barriers and facilitators to implementation.

Descriptive statistics were used to summarize quantitative survey responses with frequency and percentage for categorical variables, and with range, mean, and standard deviation for continuous variables. Variables were first grouped based on health system tracking stage, as reported in Section II, to compare data on maturity of current equity practices. Cross-tabulation was used to examine trends in the relationship between tracking stage, health system institutional characteristics, and executive reporting practices. Survey responses with at least one metric mentioned were included in the analysis.

For qualitative data (Section III and Section VI), members of the research team applied codes to free-form text responses on processes in place after disparity identification, equity metrics tracked, target and benchmark setting, and facilitators and barriers to implementing system-level metrics. Target and benchmarking setting is a common practice in quality improvement and involves the establishment of specific standards against which performance can be measured. Health systems may develop a goal or baseline for a metric and track progress over time. Data were manually coded and reviewed to identify topic areas based on keywords. We used inductive coding with some pre-defined labels for common clinical indicators (e.g., diabetes, hypertension). One team member developed a codebook to group responses to free-text questions and met with two additional team members to discuss coding and resolve discrepancies. Codes and categorization for clinical indicators (e.g., whether indicators were related to chronic disease management, preventive care, or acute care) were reviewed by two clinicians.

## Results

Of the 131 health systems invited to respond, 29 responded (22.1%) and 27 participants completed the survey (20.6%). Two health systems initiated but did not complete the survey and were excluded from the analysis.

Among the 27 participating health systems, the mean number of hospitals per system was 9.5 (range 2–27), as compared with a mean of 5.6 hospitals per system nationally (Table 1). Over one-third (37.0%) were from the Midwest, nearly one quarter from the Northeast (22.2%) and West (22.2%), and less than one quarter from the South (18.5%), as compared with nationally 26.9% from the Midwest, 22.4% from the Northeast, 18.4% from the West and 32.3% from the South. Most survey participants held leadership roles in a department within the health system dedicated to equity initiatives. Seven survey participants (25.9%) were employed by their health system’s Health Equity Department, followed by six participants from a Diversity, Equity, and Inclusion (DEI) Department (22.2%).

**Table 1:** Institutional characteristics and reporting practices^a^.

	Overall (n = 27)	
Number of hospitals in system, mean (SD)	**9.5 (6.5)**	
Number of metrics tracked, mean (SD)	**4.1 (3)**	
**Health System Region, n (%)**	**N**	**%**
Midwest	10	37.0%
Northeast	6	22.2%
West	6	22.2%
South	5	18.5%
**Department Responding to Survey, n (%)**		
Health Equity	7	25.9%
Diversity, Equity, and Inclusion (DEI)	6	22.2%
Medical/Scientific	3	11.1%
Population Health	3	11.1%
Quality/Quality Improvement	2	7.4%
Informatics/Analytics	2	7.4%
Patient Experience	1	3.7%
Other	3	11.1%
**Implementation Stage, n (%)**		
Planning Phase	7	25.9%
Early Implementation (1 year or less)	9	33.3%
Implemented for 1–2 years	7	25.9%
Implemented for 3 or more years	4	14.8%
		
**System-Level Monitoring and Reporting Practices, n (%)**		
Enterprise Dashboard/Scorecard	22	81.5%
Quality Department Dashboard	22	81.5%
Population Health Department Dashboard	18	66.7%
Operational Dashboard	11	40.7%
Ad hoc analysis	14	51.9%
Other	3	11.1%
**Data Collection Training Implemented, n (%)**		
Yes	20	74.1%
No	3	11.1%
In progress	4	14.8%
**Incentives, n (%)**		
**Have incentives (financial or otherwise) for reporting**	12	44.4%
Internal performance goals related to health inequities	8	66.7%
Insurance reimbursement for risk identification	1	8.3%
Improving employee inequities	1	8.3%
Other	2	16.7%
**Do not have incentives (financial or otherwise) for reporting**	15	55.6%
**Capture of Race/Ethnicity, n (%)**		
Self-report via registration process	27	100.0%
Race/Self-report via online portal	21	77.8%
Self-report via paper form	11	40.7%
Self-report via other form	3	11.1%
Other method	2	7.4%
Not collected	0	0.0%

^a^For certain characteristics, total percentages may be greater than 100% due to the option of selecting more than one response.

All 27 health systems indicated that they collect race and ethnicity data. All indicated they capture self-reported race and ethnicity data at the time of patient registration with the health system. More than three-quarters of participating health systems (77.8%) additionally collected race and ethnicity data via a patient-facing online portal.

In terms of reporting practices, most systems monitored health equity metrics (i.e., KPIs) via a healthcare system-level and/or enterprise-level dashboard (sometimes referred to as a health equity scorecard) (81.5%). Approximately two-thirds (66.7%) of the health systems monitored health equity-related KPIs via a system-level population health dashboard (i.e., managed by a population health team or division).

Of the 27 participants, seven (25.9%) indicated that they were still in the planning phase, nine (33.3%) were in early implementation (practices implemented for one year or less), seven (25.9%) had practices implemented for one to two years, and four (14.8%) had health equity tracking practices implemented for three years or more. Across all institutions, the mean number of metrics tracked was 4.1 (range 1–10, SD 3.0), however, this varied by implementation stage. Health systems further along in their equity tracking practices tracked fewer metrics. Across systems in the planning phase, the mean number of metrics tracked was 3.1; in early implementation the mean number of metrics tracked was 7.0; for those with practices implemented for one to two years, the mean number of metrics tracked was 2.4; for those with practices implemented for three or more years, the mean number of metrics tracked was 2.5. This change in the number of metrics tracked was most pronounced between institutions in early implementation (mean 7.0, range 1–10, SD 3.1) and institutions with practices implemented for one to two years (mean 2.4, range 1–10, SD 1.3).

Across all health systems, there were 106 metrics mentioned in free-form text fields (S1 Table). The most frequently reported metrics were related to chronic disease management (23.6%). Diabetes and blood pressure control/hypertension were the most mentioned focus areas for chronic disease management. Metrics related to preventive care were also frequently mentioned (16.0%), and common metrics in preventive care included cancer screenings, mental health screenings, well child visits, and immunizations. Other metrics were categorized as related to acute care (15.1%), patient experience (12.3%), social determinants of health (10.4%), and utilization and readmissions (9.4%). Metrics categorized as acute care varied, but mortality (multiple causes) was mentioned frequently. Across systems, there was much variation in free-text responses on targets and/or benchmarks chosen to measure progress on metrics which did not allow for categorization.

The mean number of uses of a sociodemographic filter mentioned across all metrics per healthcare system was 15.9 (range 1–61, SD 15.2). Health systems could report applying up to eight filters for each of ten possible metrics reported (includes any reporting of ‘other’). Most healthcare organizations reported using more than one filter per metric. On average per health system, more filters were used for initial or primary metrics mentioned (e.g., first, second, or third metric) than subsequent or secondary metrics (e.g., sixth, seventh, or eighth metric) (Table 2). Race/ethnic sociodemographic filters were the most utilized to evaluate and compare equity (applied 104 times, 24.2%, across all metrics mentioned), followed by sex (73 mentions, 17.0%), age (59 mentions, 13.8%), language (55 mentions, 12.8%), payer type (48 mentions, 11.2%), socioeconomic status (41 mentions, 9.6%), and gender identity (33 mentions, 7.7%) (Table 3).

**Table 2:** Average number of sociodemographic filters used^a^ by metric across health systems.

	Overall		Planning Phase		Early Implementation		Practices Implemented for 1–2 Years		Practices Implemented for 3 + Years	
	*Mean*	*SD*	*Mean*	*SD*	*Mean*	*SD*	*Mean*	*SD*	*Mean*	*SD*
First metric	3.4	1.9	2.7	2.9	4.3	1.8	3.4	2.2	2.5	1.3
Second metric	3.2	2.4	2.7	2.6	4.4	2.4	3.3	2.3	1.3	1.5
Third metric	2.5	2.4	2.4	2.8	4.4	1.9	1.0	1.5	0.8	1.0
Fourth metric	1.7	2.2	0.9	1.9	4.0	2.0	0.6	1.1	0.3	0.5
Fifth metric	1.8	2.3	0.9	1.9	3.4	2.4	1.3	2.2	0.5	1.0
Sixth metric	1.2	2.1	1.3	2.4	2.6	2.4	0.0	0.0	0.0	0.0
Seventh metric	0.9	2.0	0.0	0.0	2.8	2.7	0.0	0.0	0.0	0.0
Eighth metric	0.8	2.0	0.0	0.0	2.4	2.8	0.0	0.0	0.0	0.0
Ninth metric	0.3	1.4	0.0	0.0	1.0	2.3	0.0	0.0	0.0	0.0
Tenth metric^b^	0.0	0.0	0.0	0.0	0.0	0.0	0.0	0.0	0.0	0.0

^a^For each metric inventoried, systems could select from 0 to 8 filters provided in the survey (includes the option to report an “other” filter).

^b^If systems chose the tenth metric (“other”) the option to choose a filter was not available.

**Table 3:** Use of sociodemographic filters across systems by implementation stage^a^.

	Overall (n = 429)		Planning Phase (n = 76)		Early Implementation (n = 265)		Practices Implemented for 1–2 Years (n = 67)		Practices Implemented for 3 + Years (n = 21)	
	*n*	*% total*	*n*	*% total*	*n*	*% total*	*n*	*% total*	*n*	*% total*
Race/ethnic subgroup	**104**	**24.2%**	22	28.9%	56	21.1%	18	26.9%	8	38.1%
Sex^b^	**73**	**17.0%**	10	13.2%	51	19.2%	11	16.4%	1	4.8%
Age	**59**	**13.8%**	7	9.2%	37	14.0%	10	14.9%	5	23.8%
Language	**55**	**12.8%**	11	14.5%	30	11.3%	11	16.4%	3	14.3%
Payer type	**48**	**11.2%**	9	11.8%	30	11.3%	6	9.0%	3	14.3%
Socioeconomic status	**41**	**9.6%**	5	6.6%	32	12.1%	4	6.0%	0	0.0%
Gender identity^c^	**33**	**7.7%**	9	11.8%	16	6.0%	7	10.4%	1	4.8%
Other	**16**	**3.7%**	3	3.9%	13	4.9%	0	0.0%	0	0.0%

^a^Counts represent each mention of use of a filter for a given metric. Across each metric inventoried, systems could select from 0 to 8 filters provided in the survey (includes the option to report an “other” filter).

^b^Sex refers to biological sex assigned at birth (male/female).

^c^Gender identity refers to socially constructed identities of women, men, and gender-diverse people.

The survey asked participants to describe actions taken once a disparity is identified. Twelve participants (44.4%) reported that they had incentives (financial or otherwise) for reporting on equity, eight of whom (66.7%) had performance goals related to reducing health inequities. When a disparity was identified and reported, 12 systems (44.4%) indicated the next step would be to implement performance improvement initiatives, eight systems (29.6%) indicated that the first step would be to conduct an analysis of findings (e.g., root cause analysis, determining contributing factors), five systems (18.5%) indicated they were still planning their response, and three systems reported they, in response, they would collaborate with internal and/or external partners to address the disparity (11.1%). Responses to this question were not mutually exclusive.

Common facilitators and barriers to implementing system-level metrics that emerged across healthcare systems included strategic planning, data analytics, and resourcing (Table 4). Many systems said that integration of health equity into strategic planning or quality reporting facilitated the tracking process (44.4% and 37.0%, respectively). Others said that leadership involvement (25.9%), coordination among departments (11.1%), and the creation of a workgroup or committee dedicated to health equity (11.1%) facilitated health equity tracking practices. In an analysis of barriers to implementation, analytics/data limitations were mentioned most frequently (33.3%). Examples included issues with data capture, integration, and integrity. Other health systems commonly mentioned barriers related to resourcing, which included competing organizational priorities (11%), financial limitations (11%), and workforce shortage and retention issues (7%).

**Table 4:** Reported facilitators and barriers to implementing system-level metrics.

Topic Areas	Sample Response(s)
***Facilitators***	
Integration into strategic plan	*“Advancing Health Equity is a key priority in the organizational strategic plan”*
	*“Added equity as a priority in the health system strategic plan with measurable objectives”*
Integration into quality reporting	*“Integration into quality reporting framework”*
	*“Integrating into our annual quality plan’*
Leadership involvement	*“C-Suite level position focused on work”*
	*“Leadership support and buy-in”*
Coordination among departments	*“Working closely with quality and safety, community health and patient experience”*
Work group or committee creation	*“There is an Health Equity Task Force led by our CEO with cross representation from Quality, Nursing, HR, Population Health…”*
	*“Multi-stakeholder work group”*
Clinician involvement	*“Provider involvement; nursing involvement; care coordination involvement”*
***Barriers***	
Analytics Limitations	*“Accurate data to make decisions - as you dive deeper into the data, you find more questions and additional need for data/research.”*
	*“Data acquisition, integrity, stewardship”*
Coordination Issues	*“We need to align and coordinate our health equity reporting across QI and population health…”*
	*“Large system with multiple business units...interoperability of systems.”*
Lack of Guidance on Equity Tracking	*“Awareness of the importance and impact Accountability for outcomes”*
	*“Lack of guidance from our typical sources, e.g., AHRQ and NQF on how to even begin addressing the data need”*
Competing Priorities	*“We are challenged by competing priorities”*
	*“Prioritization of this work and the resources needed to complete it”*
Financial Issues	*“Lack of adequate funding”*
	*“Reimbursement for innovative interventions”*
Workforce/Retention	*“Many priorities. Lack of resources to address disparities. e.g., we are aware of disparities as regards colon cancer screening in our population. We already have a backlog of cases to be scheduled/ not enough GI doctors to perform given some recent resignations.”*
Strategic Alignment	*“There are so many health equity metrics to consider that we have been charged by our health system CEO to select 1–2 measures to focus on initially; consequently our task is to align the selected measures with our system’s other strategic QI and population health priorities.*
Cultural Issues	*“Cultural”*

### Limitations

This was a voluntary survey. Findings are not representative of all health systems in the U.S. and likely represent early adopters of equity practices. We would expect DHEI network health systems with a greater focus on equity to self-select to respond as early adopters (i.e., non-respondents may be less likely to be measuring health equity at this time) given that the inclusion of metrics focused on identifying disparities in care is relatively new practice. However, as this research focuses on current health equity tracking practices, we believe our findings from the health systems that chose to participate provide meaningful insights that can inform future efforts across the industry. Data were self-reported; thus, there may be a certain degree of bias, uncertainty, and variation in results, including but not limited to, the participant’s definition of “entire health system.” Additionally, with a small sample size we did not test for statistical significance and relied entirely on a descriptive analysis. The qualitative analysis relied on short, free-form text responses; full context on barriers and facilitators to equity tracking could not be captured. This could be explored further with focus groups or individual respondent interviews. Further, given that this survey was focused on assessing the tracking of health equity gaps and disparities in outcomes, we did not assess variation in quality improvement dashboards across organizations and how the content and functionality of these dashboards may differ. Future work should include examination of these differences as they could inform efforts to develop comprehensive best practice standards for the industry.

## Discussion

We identified commonalities in how health equity is measured and monitored, yet substantial variation. Many health systems have a health equity or DEI department dedicated to this work and shared that they use executive-level dashboards for monitoring and reporting. Across health systems, metrics related to chronic disease and preventive care were commonly tracked and race/ethnicity was often used as a parameter to identify differences between groups. Respondents also shared that integrating health equity into a strategic plan or quality reporting and involving leadership can support the implementation of health equity work. Despite these shared attributes, there is variation in how health equity is measured and monitored, through the metrics and sociodemographic filters applied, the targets and benchmarks set to achieve equity goals, and the overall implementation stage for tracking equity at the executive-level. There is a crucial need and opportunity to develop best practices and a healthcare industry standard for health equity related metrics that can be implemented across health systems in the U.S. It will be important for the long-term sustainability of health equity initiatives to have consistent executive visibility and strategic support tied to these metrics.

To our knowledge, this study is the first to assess real-world practices to systematically track and evaluate health equity gaps and/or disparities in health outcomes across health systems in the U.S. at the executive level. While there have been some efforts described in the literature to create health equity-focused dashboards in clinical contexts [14], including in the emergency department [15,16], in pediatrics [17,18], and at the population-level within a designated geography [19–21], little is known about standard efforts underway across U.S. health systems at the executive level [22]. While most health systems in our research reported using dashboards for system-level monitoring and reporting practices, there was variation in the metrics being prioritized and the standards being set to monitor progress over time.

In addition to a lack of standards for capturing data on key variables needed to assess health equity, there is a dearth of standard measures for assessing performance in improving health equity [23,24]. Indices have been developed by different entities [25], but without standard measures for assessing performance both within and across health systems, it remains a challenge to create accountability [24]. This is central to a shared executive-level accountability framework that can be adopted across hospitals, driving societal change and improvement. Innovative approaches, including adaptation of existing indexes intended to quantify disparities and/or the use of predictive analytics, warrant further examination to identify pros and cons to different approaches to inform best-practice recommendations [26,27]. While most health systems were either in the planning or early implementation phase, our finding that the number of metrics tracked varied with the implementation phase is important. This may be indicative of challenges faced in the implementation process that tempered ambitious plans resulting in the pivot towards a more feasible approach. Participants shared that integrating equity into strategic planning and quality reporting were facilitators to tracking, yet analytics and data limitations were the most frequently noted barriers to equity tracking. At the time of this publication there is no national standard for capturing and reporting key sociodemographic data needed to assess equity gaps, and the guidance provided by regulatory bodies and organizations is inconsistent across agencies [23,24]. Standard guidance could help organizations set up the technical infrastructure needed to collect and report on this data.

More guidance is also needed to develop industry standard best practice guidelines for equity goal setting and measurement. Our findings suggest that health systems are following some guidance from existing frameworks, but not consistently given that these frameworks and requirements often do not include details as to how to operationalize them. For example, within the CMS Framework for Health Equity [4], many health systems align with the priority area of expanding sociodemographic data collection or addressing disparities, but no details are provided as to how to achieve this and ensure data integrity, accuracy, and validation. Guidance may be most effective if it comes from healthcare quality and/or regulatory agencies within healthcare systems, given that health systems’ quality and safety teams often play an active role in operationalizing regulations to implement health equity metrics, and as such, have an in-depth understanding of the challenges and realities of such efforts. Widespread, consistent monitoring of metrics across health systems can help address equity gaps over time; monitoring these data allows researchers and practitioners to analyze what is helping or hindering progress so they can work to close these gaps. Our analysis of reported metrics suggests that there are potential areas where regulatory agencies could focus their efforts when developing new standards, like underscoring the collection of standard race/ethnicity data to identify disparities in chronic disease management. One example of prescriptive regulatory action intended to standardize measurement can be found in a recent mandate from the California Department of Health Care Access and Information (HCAI) Hospital Equity Measures Reporting Program which requires California hospitals to collect and analyze specific health equity data and publish a health equity report to the hospital’s and HCAI’s websites annually beginning September 2025 [28]. Some organizations have started to work together to collectively define industry standards, like the Institute for Healthcare Improvement (IHI) Leadership Alliance [29] as well as the IHI Health Equity Accelerator. The goal of these efforts is to inform the development of recommendations and guidance to eventually define best practices for health equity measurement across institutions.

As an accountability mechanism, national agencies can require that healthcare systems incorporate equity components in reporting on healthcare quality. As an example, the Joint Commission now requires that health systems identify health care disparities in their patient populations using quality and safety data [9]. They could also require the development and submission of an executive strategy for mitigating equity gaps. These executive strategies could incorporate elements of an existing equity guide, like the CMS Framework for Health Equity [4]. Furthermore, routine quality checks of patient reported demographic data is essential, including ongoing education for clinicians, providers, and front-line staff on the role of demographic data in quality improvement [30].

## Conclusions

There is momentum around addressing equity across healthcare systems; however, results from this survey suggest that systems are in the initial stages of implementing equity measures and tracking progress on disparity reduction, and there is variation in how health systems are approaching it. This research suggests a critical and timely need for collaboration between health systems and national and state agencies on healthcare quality and equity. A collaborative approach between healthcare delivery organizations and healthcare agencies will allow for co-development of flexible best practices that provide a framework to evaluate whether health system efforts are meaningfully impacting outcomes and to work toward improving gaps in equity. Health system executives, as representatives of their respective organizations, are best positioned to set these standards when working in collaboration with each other and regulatory agencies. Agencies on healthcare quality and equity can expand on work that has already been done within health systems, as identified in this survey, to best reflect the priorities of the healthcare delivery system.

## Supporting information

S1 Appendix Health equity survey for health systems. (ZIP)

S1 Table Health equity metrics reported. (ZIP)
