# Supplementary figures and images for: Measure what matters: A survey-based examination of health equity tracking and measurement practices across healthcare systems in the United States
Source: PLoS One. 2025 May 21;20(5):e0323381. doi: 10.1371/journal.pone.0323381 (PMC12094744; doi:10.1371/journal.pone.0323381)

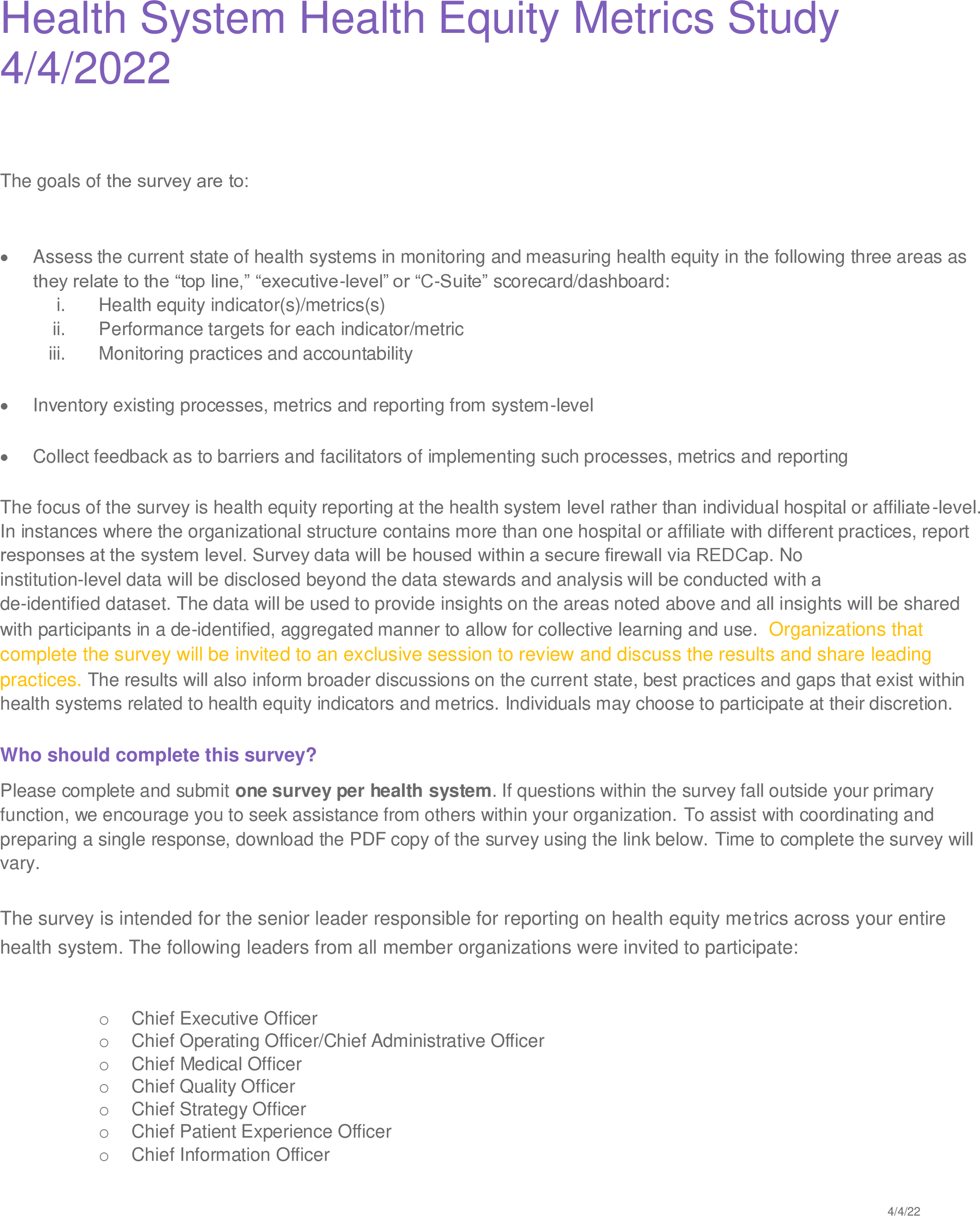

Supplement: S1 Appendix — (ZIP) [file pone.0323381.s001.zip › PACE Corrected/S1_Appendix.tif]

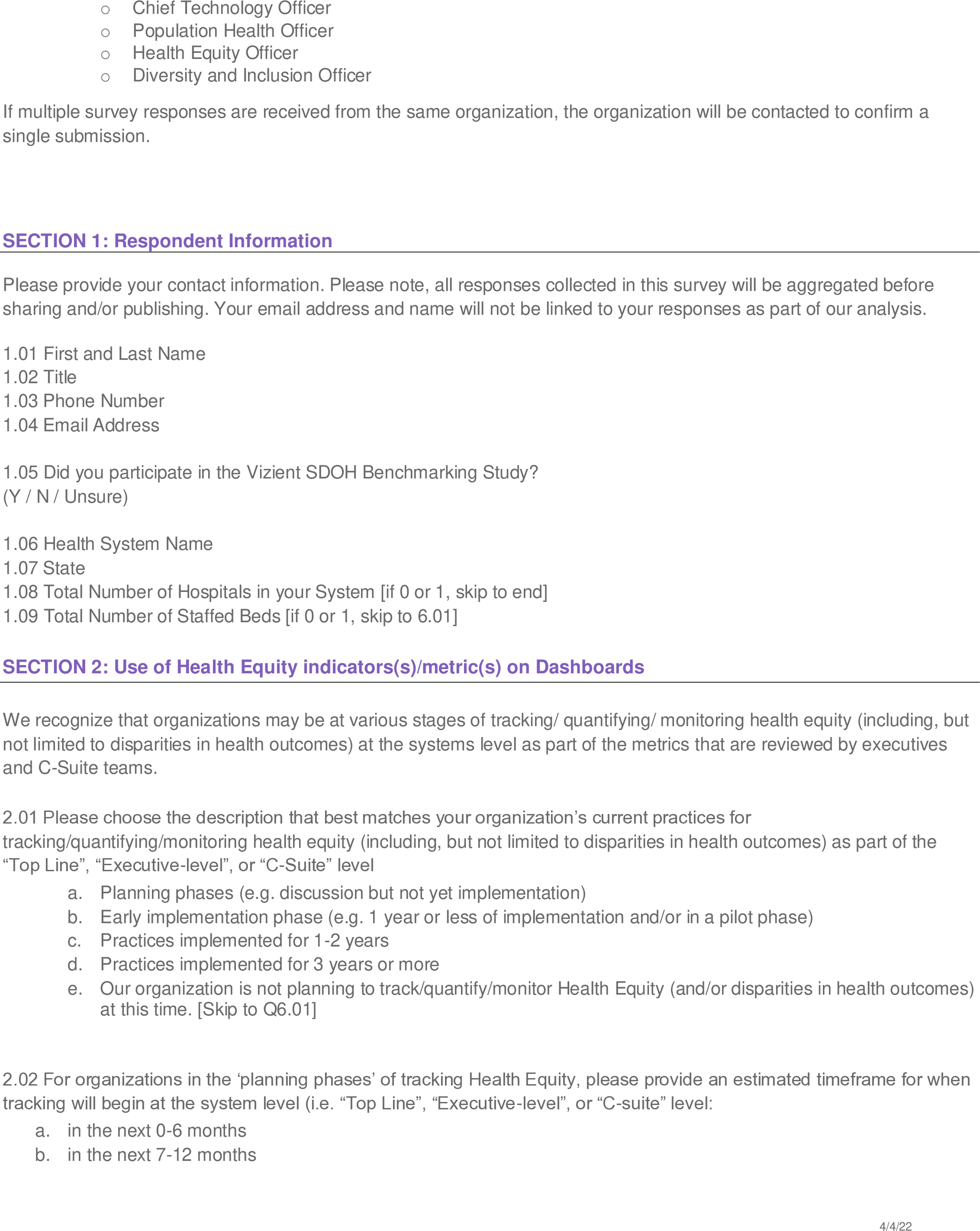

Supplement: S1 Appendix — (ZIP) [file pone.0323381.s001.zip › PACE Corrected/S1_Appendix.tif]

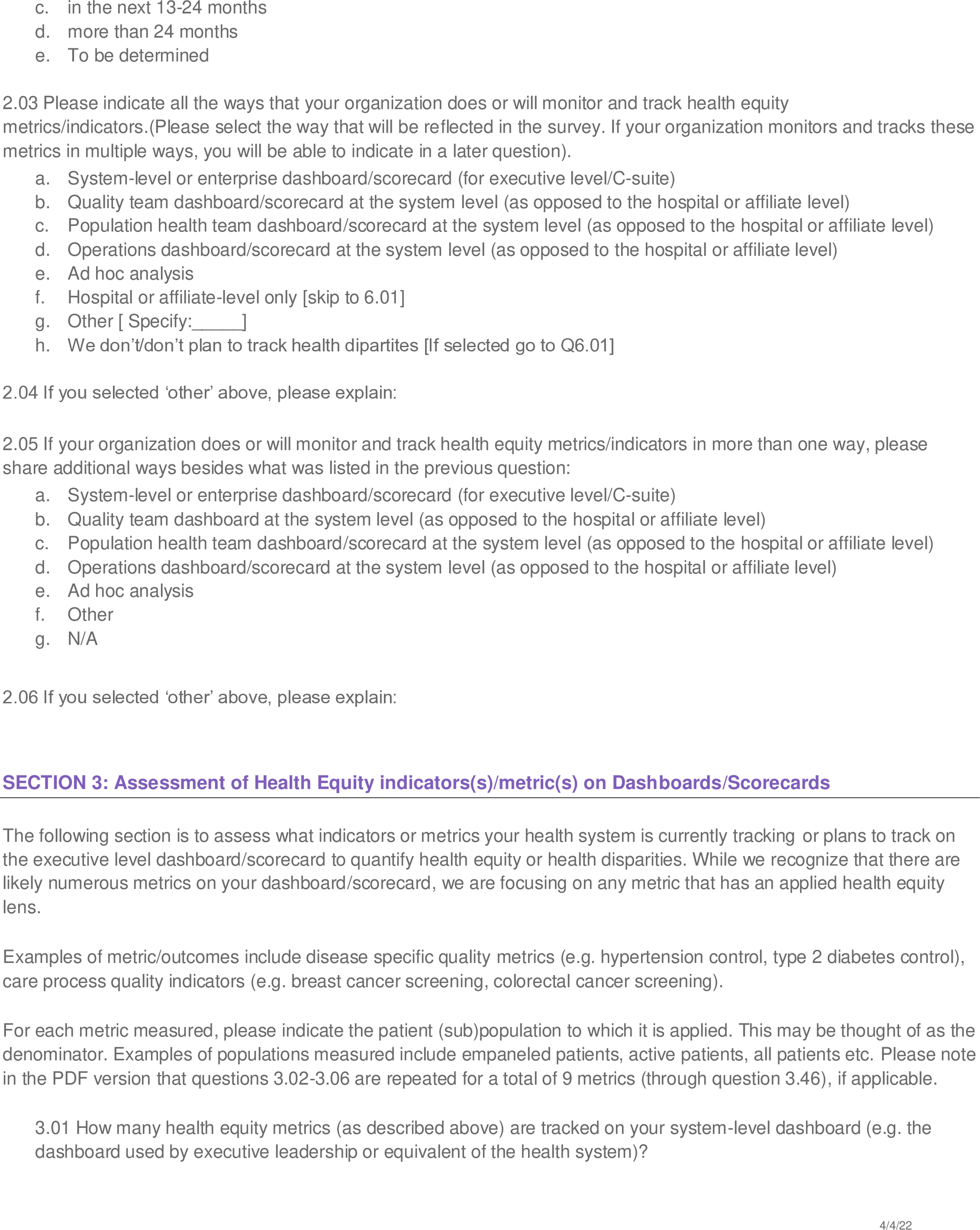

Supplement: S1 Appendix — (ZIP) [file pone.0323381.s001.zip › PACE Corrected/S1_Appendix.tif]

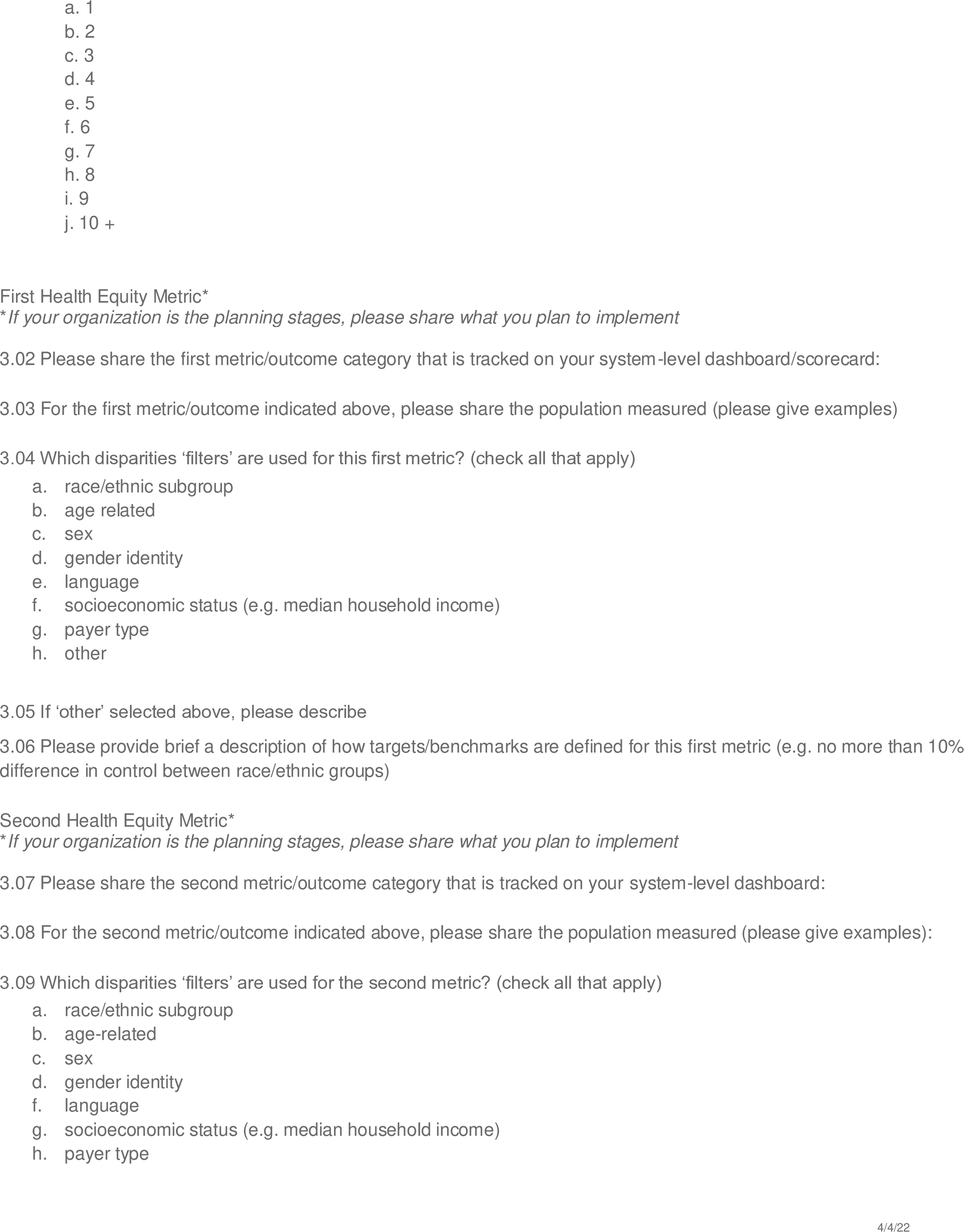

Supplement: S1 Appendix — (ZIP) [file pone.0323381.s001.zip › PACE Corrected/S1_Appendix.tif]

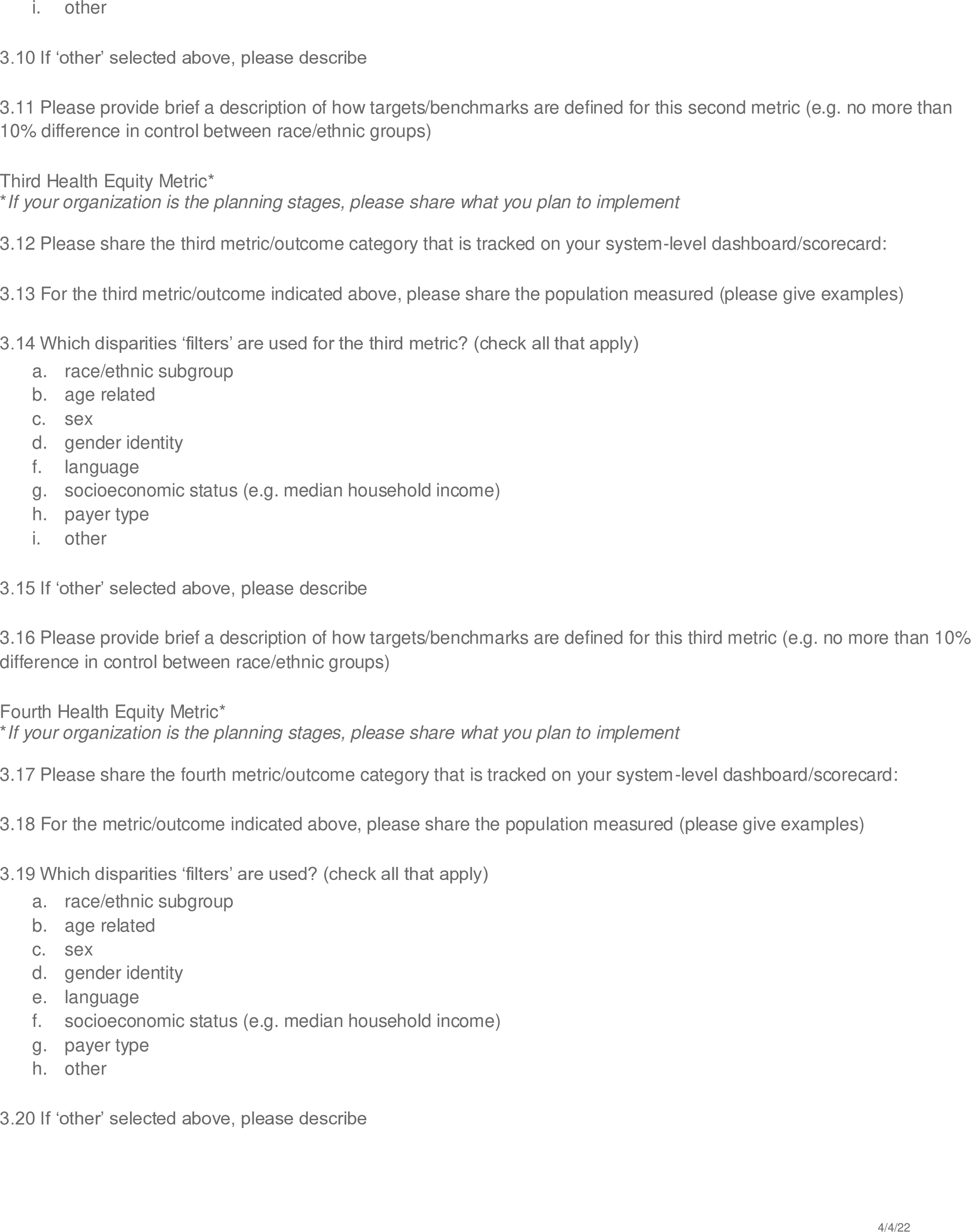

Supplement: S1 Appendix — (ZIP) [file pone.0323381.s001.zip › PACE Corrected/S1_Appendix.tif]

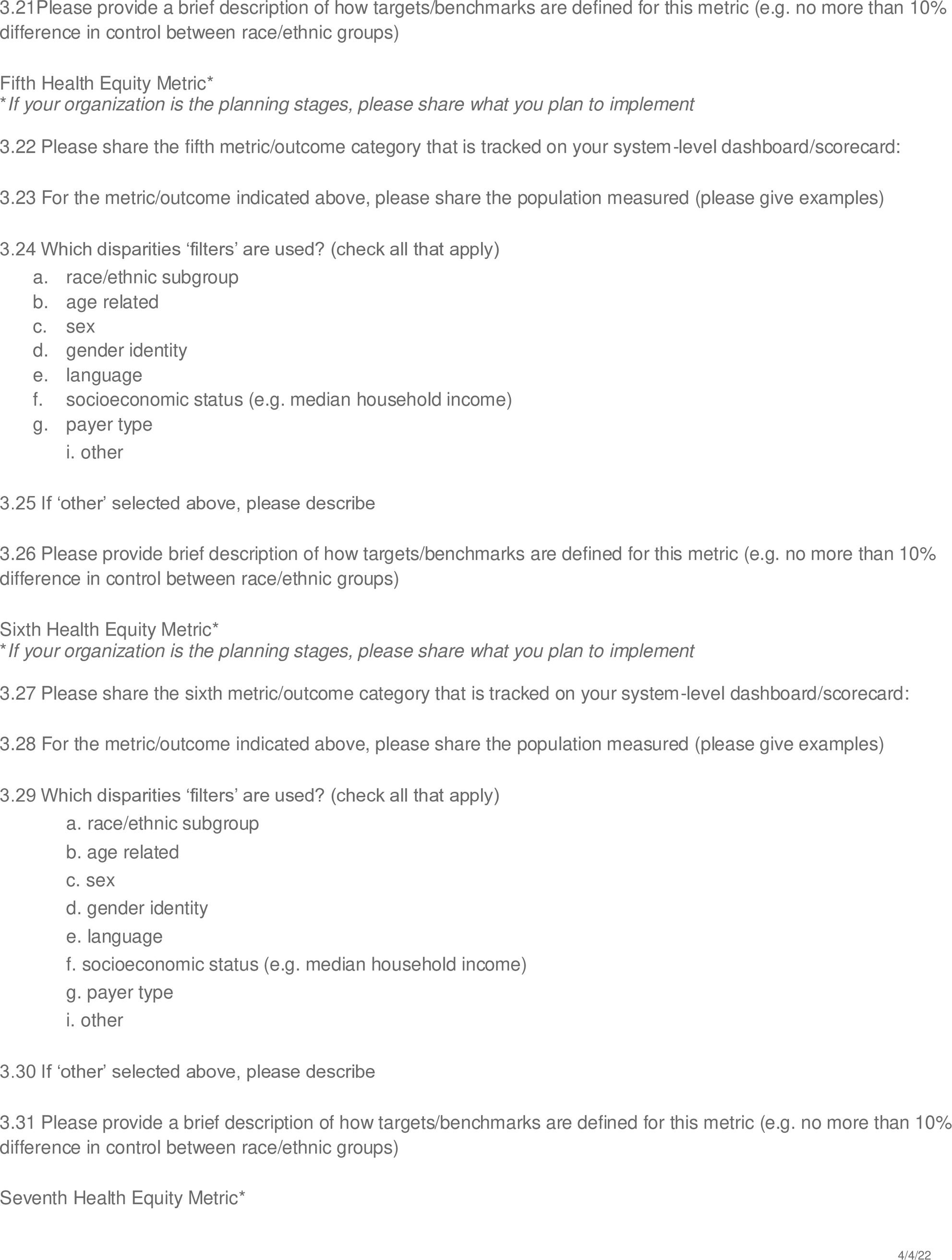

Supplement: S1 Appendix — (ZIP) [file pone.0323381.s001.zip › PACE Corrected/S1_Appendix.tif]

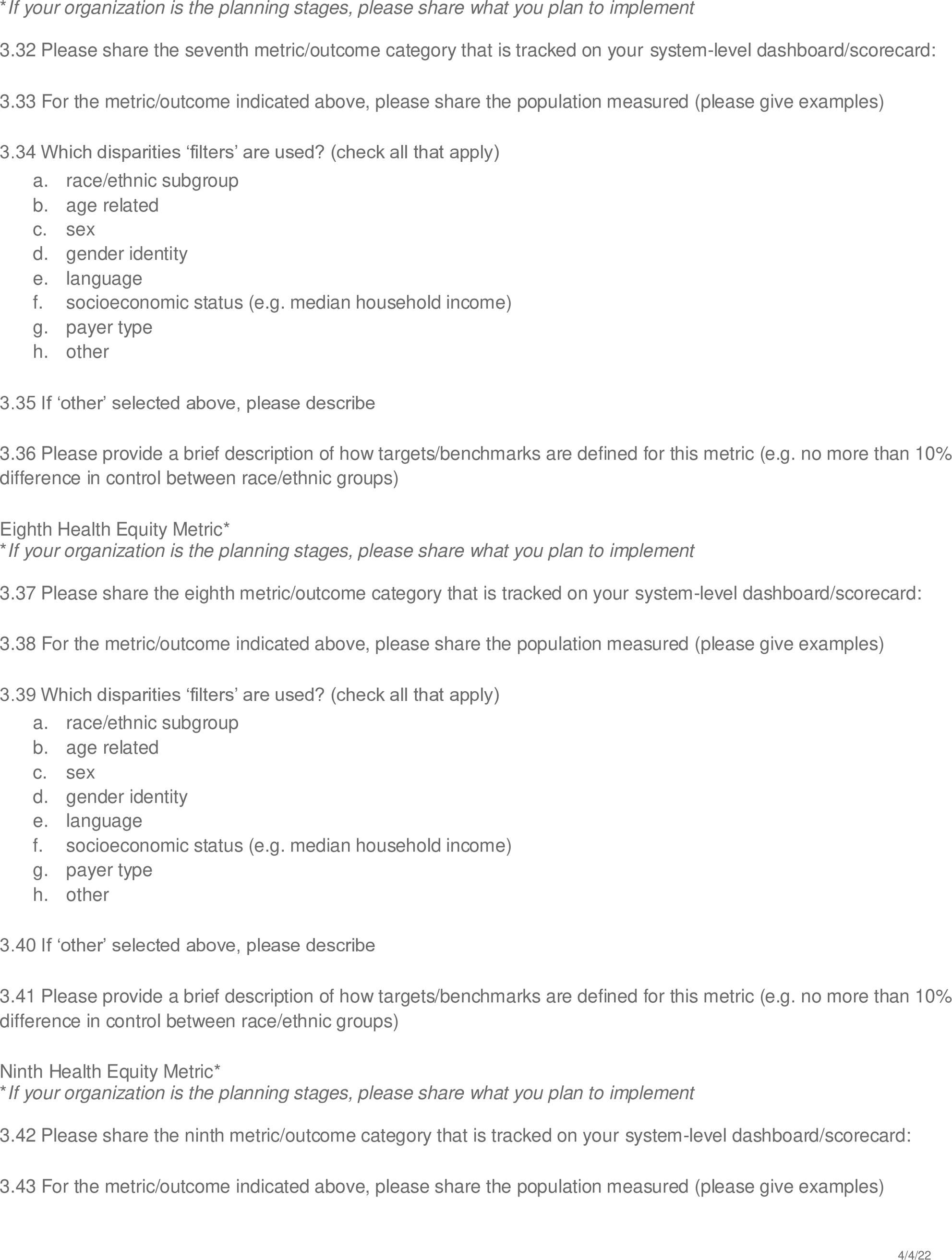

Supplement: S1 Appendix — (ZIP) [file pone.0323381.s001.zip › PACE Corrected/S1_Appendix.tif]

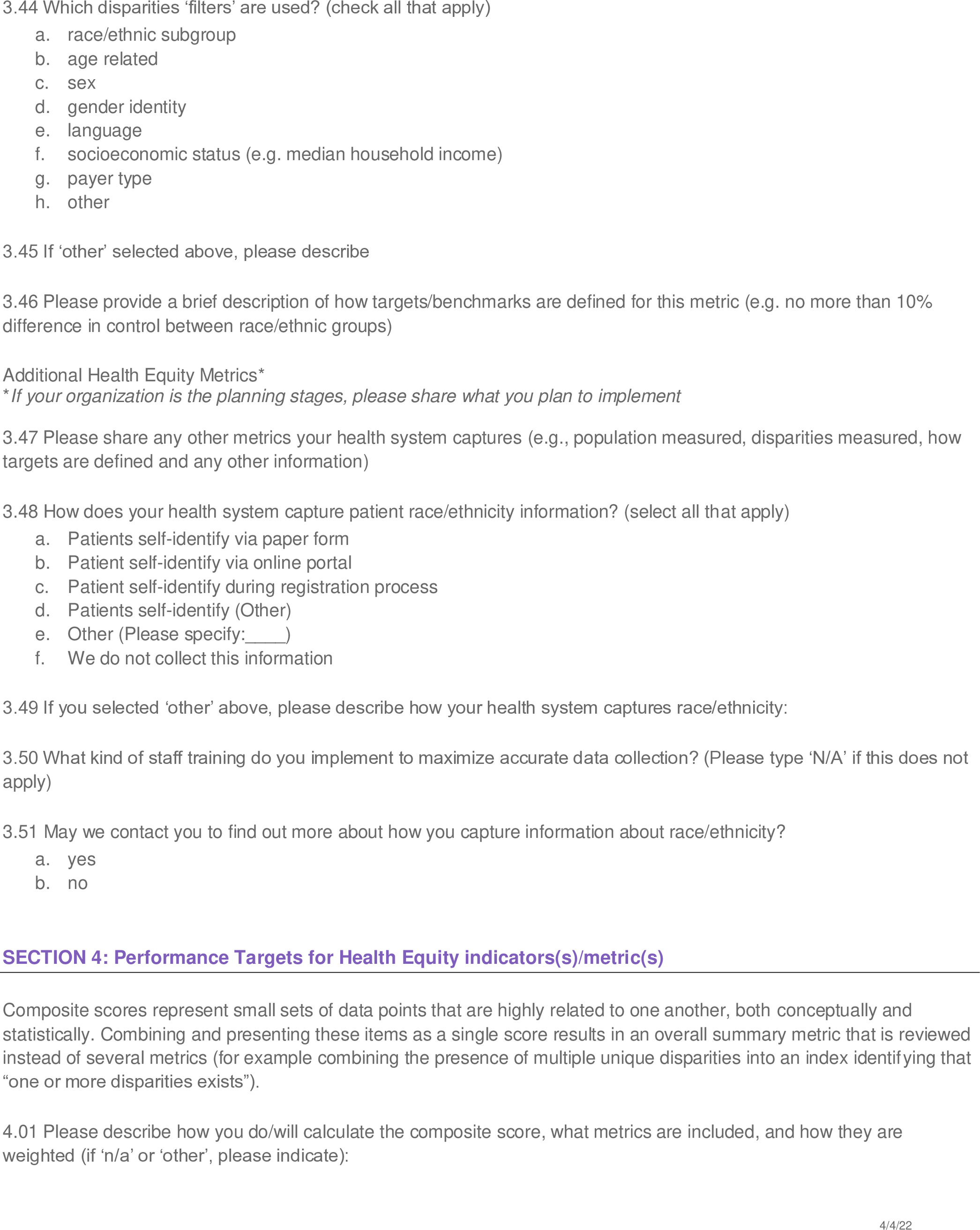

Supplement: S1 Appendix — (ZIP) [file pone.0323381.s001.zip › PACE Corrected/S1_Appendix.tif]

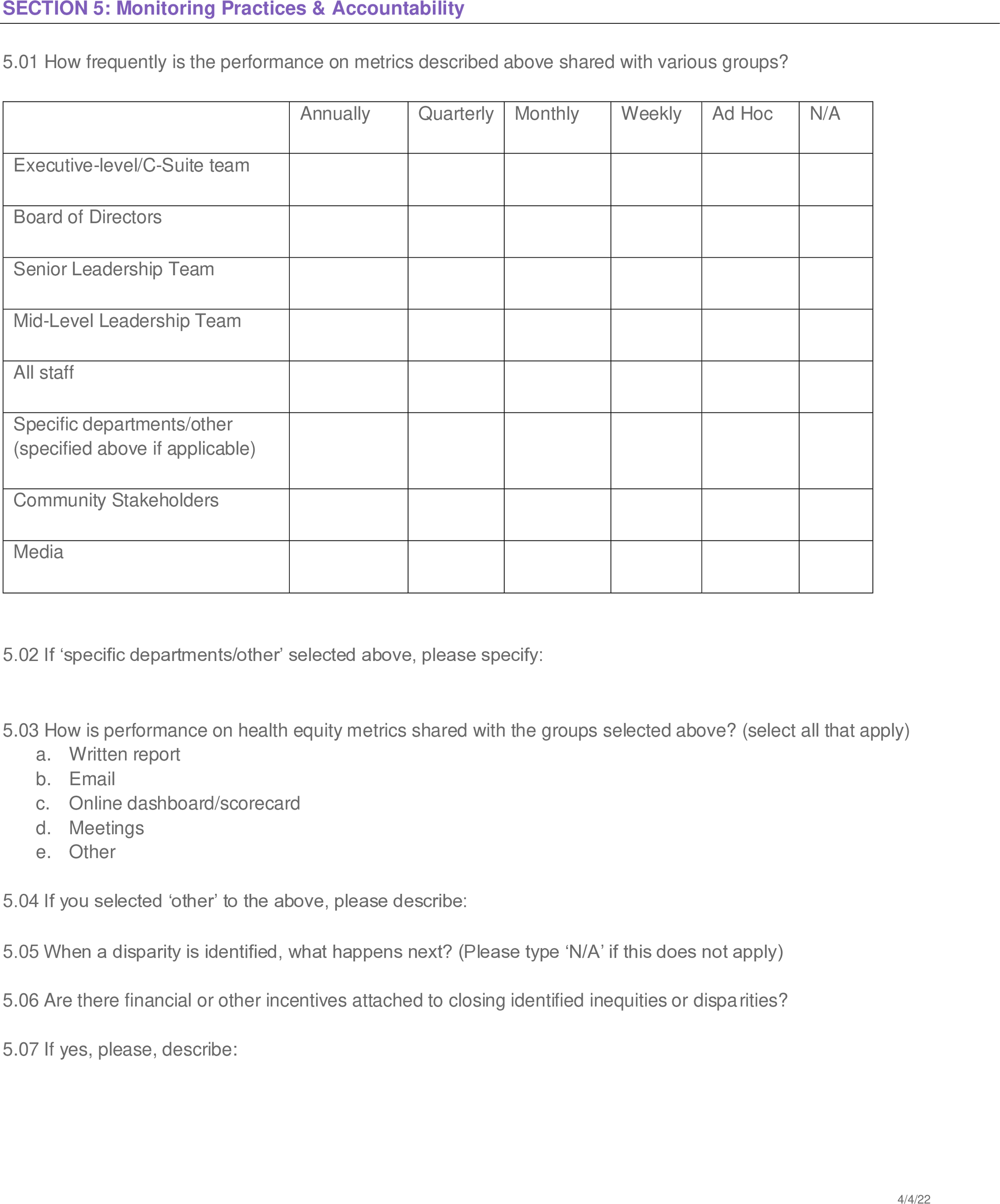

Supplement: S1 Appendix — (ZIP) [file pone.0323381.s001.zip › PACE Corrected/S1_Appendix.tif]

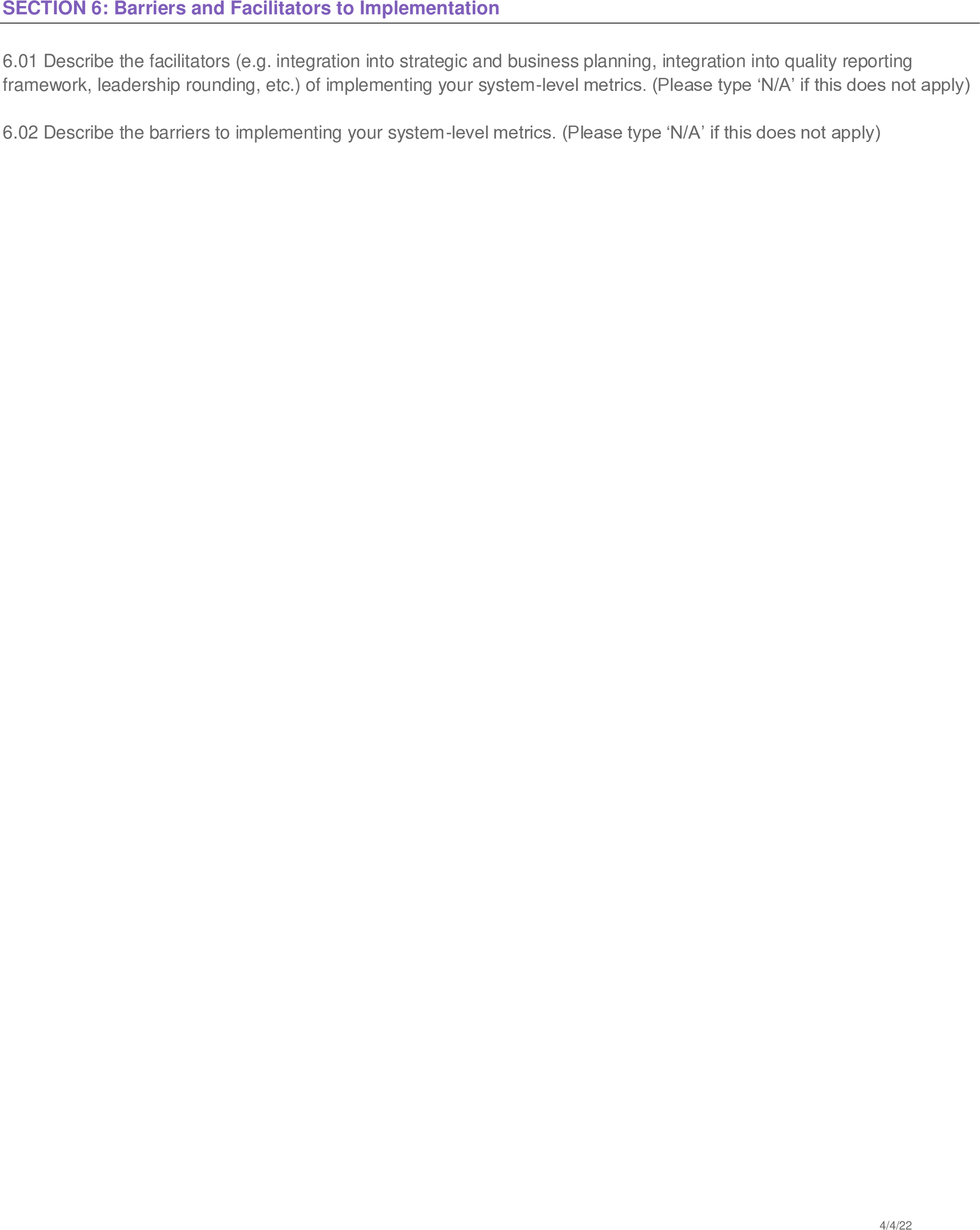

Supplement: S1 Appendix — (ZIP) [file pone.0323381.s001.zip › PACE Corrected/S1_Appendix.tif]

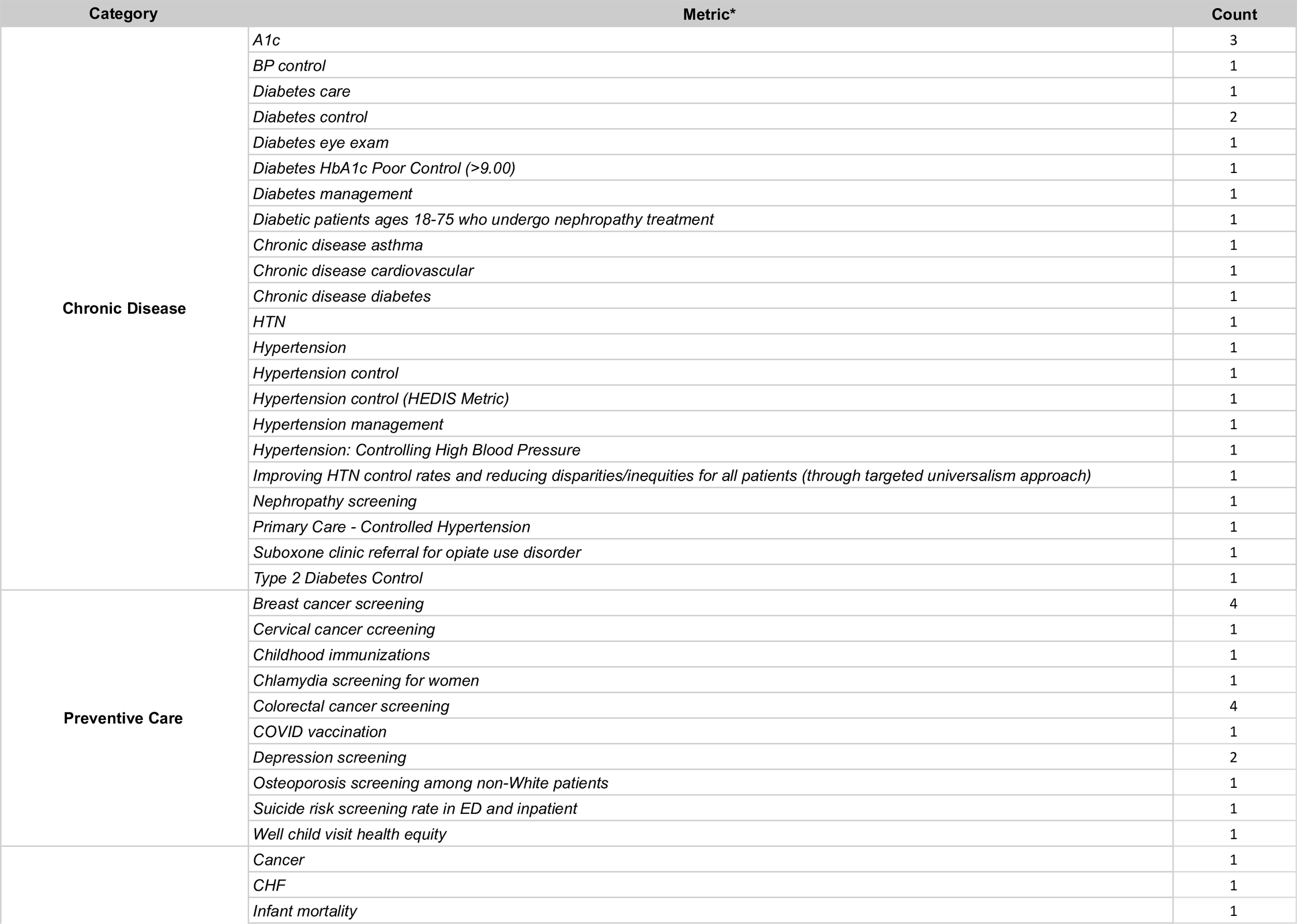

Supplement: S1 Table — (ZIP) [file pone.0323381.s002.zip › PACE Corrected/S2_Table.tif]

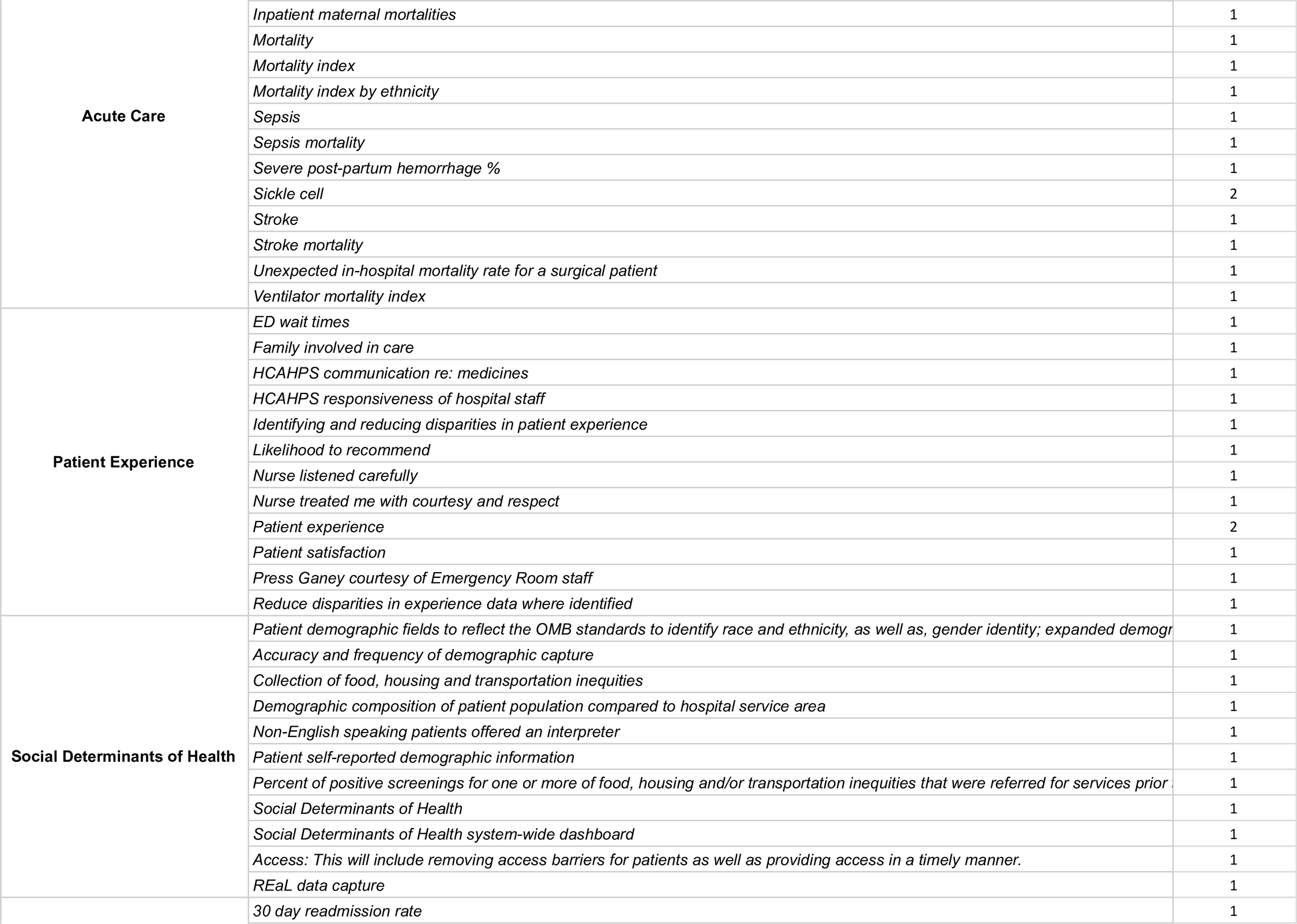

Supplement: S1 Table — (ZIP) [file pone.0323381.s002.zip › PACE Corrected/S2_Table.tif]

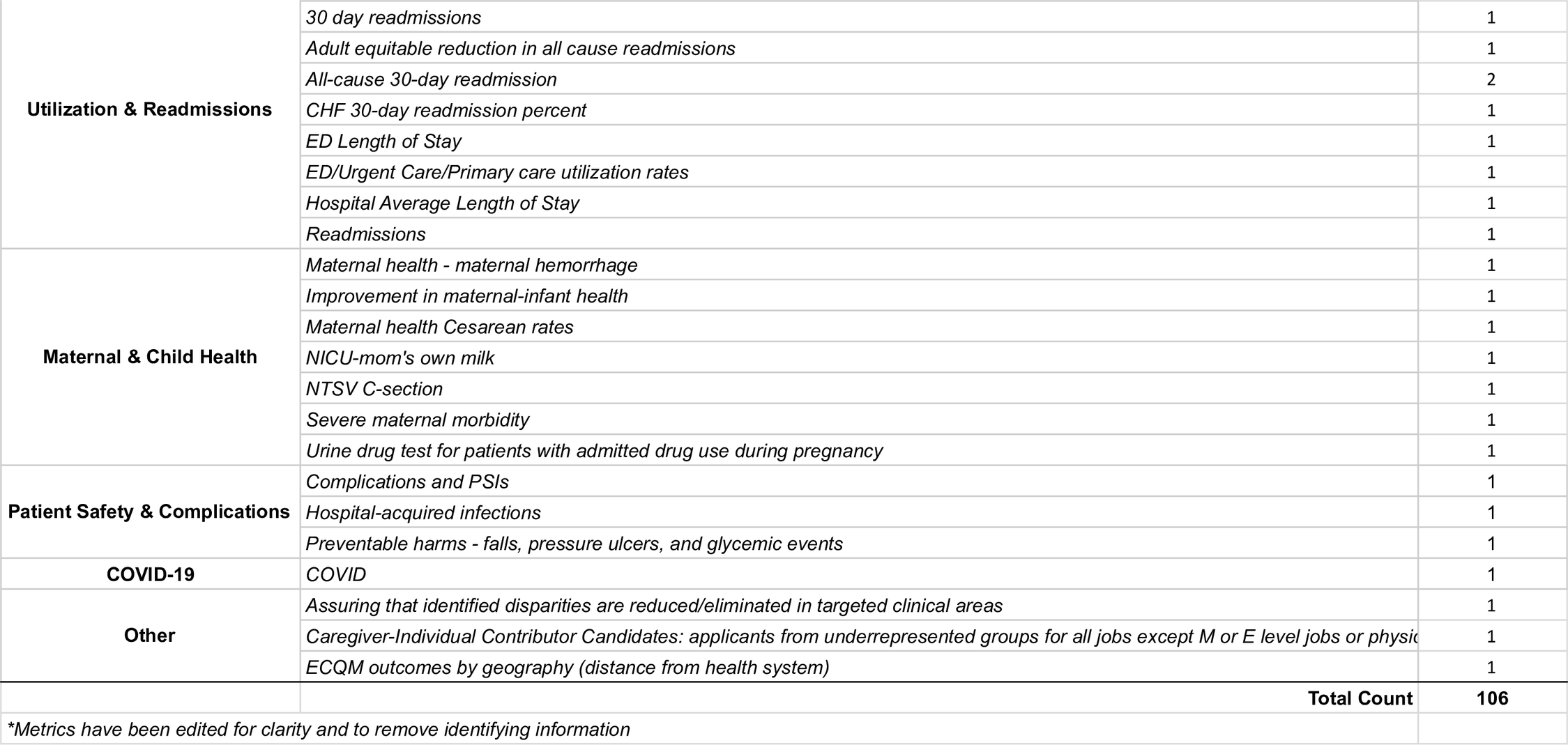

Supplement: S1 Table — (ZIP) [file pone.0323381.s002.zip › PACE Corrected/S2_Table.tif]
